# Variation in Terpene Profiles of *Thymus vulgaris* in Water Deficit Stress Response

**DOI:** 10.3390/molecules25051091

**Published:** 2020-02-28

**Authors:** Atiyeh Mahdavi, Parviz Moradi, Andrea Mastinu

**Affiliations:** 1 Department of Biological Sciences, Institute for Advanced Studies in Basic Sciences (IASBS), Zanjan 45195, Iran; atiyeh.mahdavi@gmail.com; 2 Zanjan Agricultural and Natural Resources Research & Education Centre, AREEO, Zanjan 45195, Iran; 3 Department of Molecular and Translational Medicine, University of Brescia, 25123 Brescia, Italy

**Keywords:** volatiles, drought stress, *Thymus vulgaris*, Adaptation mechanisms, physiology, tolerant and sensitive plants

## Abstract

Thyme (*Thymus* spp.) volatiles predominantly consisting of monoterpenes and sesquiterpenes, serve as antimicrobial, antiseptic and antioxidant in phytomedicine. They also play a key role in plants as secondary metabolites via their potential role against herbivores, attracting pollinators and abiotic stress tolerance. Plant volatiles are affected by different environmental factors including drought. Here, the effect of prolonged water deficit stress on volatile composition was studied on the sensitive and tolerant thyme plant cultivars (*T. vulgaris* Var. Wagner and *T. vulgaris* Var. Varico3, respectively). Volatile sampling along with morpho–physiological parameters such as soil moisture, water potential, shoot dry weight, photosynthetic rate and water content measurements were performed on one-month-old plants subsequent to water withholding at 4-day intervals until the plants wilted. The tolerant and sensitive plants had clearly different responses at physiological and volatile levels. The most stress-induced changes on the plants’ physiological traits occurred in the photosynthetic rates, where the tolerant plants maintained their photosynthesis similar to the control ones until the 8th day of the drought stress period. While the analysis of the volatile compounds (VOCs) of the sensitive thyme plants displayed the same pattern for almost all of them, in the tolerant plants, the comparison of the pattern of changes in the tolerant plants revealed that the changes could be classified into three separate groups. Our experimental and theoretical studies totally revealed that the most determinant compounds involved in drought stress adaptation included α-phellandrene, O-cymene, γ-terpinene and β-caryophyelene. Overall, it can be concluded that in the sensitive plants trade-off between growth and defense, the tolerant ones simultaneously activate their stress response mechanism and continue their growth.

## 1. Introduction

Secondary metabolites such as volatile compounds (VOCs) are synthesized by various plant organs and play many important functions for the protection of plants under different ecological interactions and stressful conditions including abiotic stresses and exposure to predators and/or pathogen, etc. [1,2,3,4,5,6]. VOCs have low molecular weight and are categorized into four chemical classes: (i) terpenoids (ii) amino acid derivatives (iii) benzenoid and phenylpropanoid compounds and (iv) fatty acid derivatives [7]. Plants produce VOCs either constitutively (CVOCs) or in response to different biotic or abiotic stresses (IVOCs) [7,8,9]. Isoprenoids are the largest and most diverse compounds of plant volatiles that are called terpenes or terpenoids and over 20,000 isoprenoids have been recorded so far [10].

*Thymus vulgaris* L. (thyme) is an aromatic plant and member of the Lamiaceae family. This medicinally important herb is distributed in various areas of the globe, particularly in the Mediterranean regions [11]. Different species of thyme have been used broadly in food, cosmetics and pharmaceutical industries [12]. Several manuscripts have been published in recent years to qualitatively and quantitatively determine the metabolome of thyme plants [13,14]. For 162 taxa of *Thymus* genus, almost 360 different VOCs have been recorded. The largest amounts of these aroma compounds belong to monoterpenes and sesquiterpenes [14].

Plant volatiles are significantly altered by biotic and abiotic stresses [15]. Previous studies established the impact of abiotic stresses including temperature [16], light [17], water [18] [19,20], salt [21] and oxidative stresses [22] on VOCs. These stresses generally increase the emission of a wide range of terpenes, including isoprene [23], monoterpenes [24] and sesquiterpenes [25] in the stress-exposed plants. Nevertheless, there are some studies that indicated no influence of stress on the release of VOCs, such as salt stress on poplar [26] and moisture stress on isoprene [27]. Previous investigations on thyme have indicated that volatile composition is affected by environmental factors including drought [28,29]. Despite the documented efficacy of volatiles in increasing plant adaptation to different stresses including water depletion, there is limited information concerning detailed mechanisms of VOCs functions in plants’ responses to stress [8,30,31].

This study aimed to monitor and compare the monoterpenes and sesquiterpenes of thyme in drought-tolerant (*Thymus vulgaris* Var.Varico3) and drought-sensitive populations (*T. vulgaris* Var. Wagner) during long-term water stress.

## 2. Results and Discussion

### 2.1. Physiological Parameters Affected by Long-Term Drought Stress

**Figure 1:**
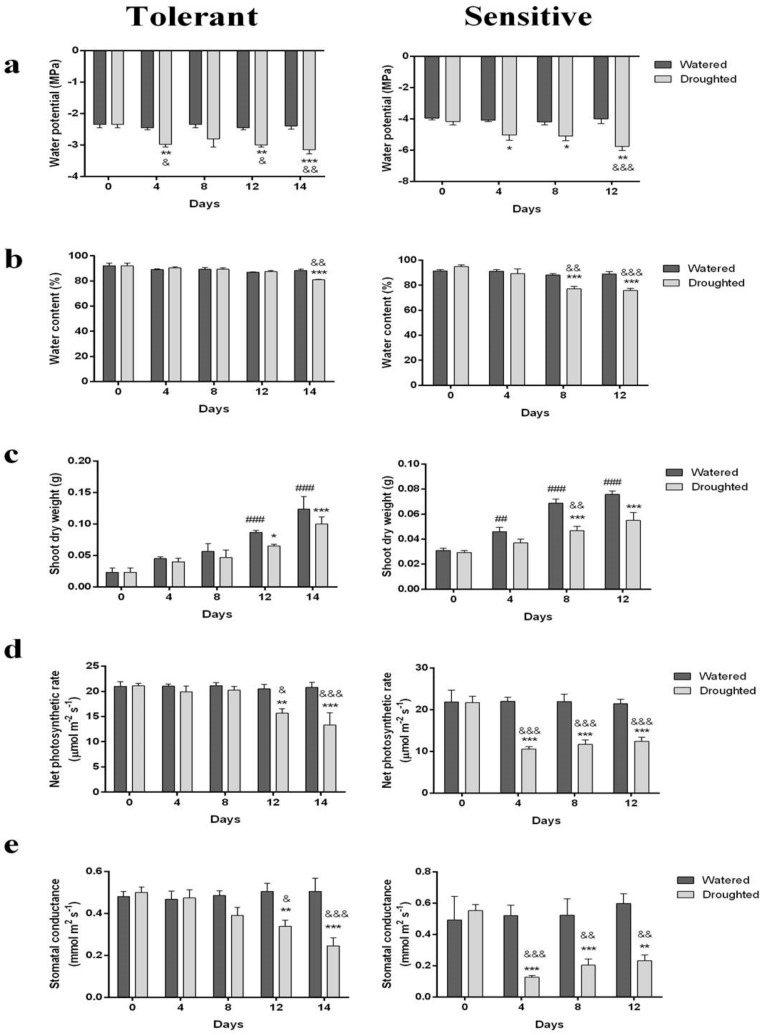
The effects of drought stress on the physiological parameters in the sensitive (right) and tolerant (left) thyme plants: (**a**) the leaf water potential (MPa); (**b**) water content (%); (**c**) shoot dry weight (g); (**d**) net photosynthetic rate (µmol m^−^^2^ s^−1^); (**e**) stomatal conductance (mmol m^2^ s^−1^). ^*^ *p* < 0.05 vs. day 0 of Droughted group; ^**^ *p* < 0.01 vs. day 0 of Droughted group, ^***^ *p* < 0.001 vs. day 0 of Droughted group, ^##^ *p* < 0.01 vs. day 0 of Watered group; ^###^ *p* < 0.001 vs. day 0 of Watered group; ^&^ *p* < 0.05 Droughted vs. Watered group; ^&&^ *p* < 0.01 Droughted vs. Watered group; ^&&&^ *p* < 0.001 Droughted vs. Watered group. Data are expressed as the mean ± S.E.M. Two-way ANOVA followed by the Sidak’s multiple comparisons test was executed.

In the present study, the investigated physiological parameters included water potential, water content, shoot dry weight and net photosynthesis rate that were affected in both populations and there was a significant difference between them in two groups (Figure 1). In the tolerant plants, water potential was reduced on the 4th day compared to the control group and was almost around −3 MPa until the end of the stress period, except for a slight increase on the eighth (Figure 1a). The potential finally reached a value slightly less than −3 MPa at the end of stress (on the 14th). In the sensitive thyme plants, while water potential continuously reduced during the water stress period compared to the control one (from initially −4 to around −5.8 MPa at the end of the stress exposure), it was almost constant from the 4th till the 8th day in the experimental plants and then declined from the 8th day onward (Figure 1a). Totally, it is seen that the tolerant thyme plants had a water potential higher than the sensitive ones.

Roots of many plants are often exposed to severe changes of water potential in their surrounding environments. Details of the adaptability and resistance of root cells to drought stress are not completely known by researchers. The results of previous studies have shown that low water potentials (ψ_w_) induced changes in plant root composition, and root growth is inhibited at low ψ_w_. However, it is much less inhibited than shoot growth. Water deficient stress resulted in decreases in root growth, root biomass, and lateral root number, and considerably damaged the root apical structure. The relative amounts of volatiles such as monoterpenes and sesquiterpenes are altered with drought stress and their ratio also changes in the root tip. In plants like maize (*Zea mays* L.) retention of root elongation preferentially happens toward the root apex along with significant increases in some amino acids concentration, especially proline which increases dramatically in the growing region of plant primary roots at low water potentials (ψ_w_). Several reports revealed that drought stress-induced modifications in the membrane lipid composition of root cells might affect membrane mass and the degree of lipid fluidity that would finally decrease water permeability of the plasma membranes and help retain cell turgidity. Some genes have been detected in root tissues of some plants (e.g., Soybean, maize, oat) that were related to carbohydrates, key hormones, lipids and cell wall-related metabolism and help plant achieve drought tolerance capacity. The expression patterns of these genes changed differentially during drought stress exposure [32,33,34].

The results obtained from the comparison of the water content in the tolerant species and control ones revealed no significant difference between them until the 4th day. On the contrary, water content dropped (from initially 94% value) to 85% on the 8th day, 75% on the 12th day and finally 65% on the 14th day in the tolerant populations compared to the control plants (Figure 1b). In contrast, the sensitive plants had 94% water content initially which dropped to 78% on the 8th day and was constant until the 12th day.

The plant shoot dry weight measurements revealed that the dry weight of the tolerant thyme plants sharply increased during the stress period around fivefold and ranged from 0.02 g at the beginning of water withholding to 0.10 g at the end of stress exposure (Figure 1c). However, it was always slightly lower than the control group, especially on the 12th day. The dry weight of the sensitive plant shoots increased each 4 days. Despite these increases, there was no significant difference between the 8th and 12th days (Figure 1c).

**Figure 2:**
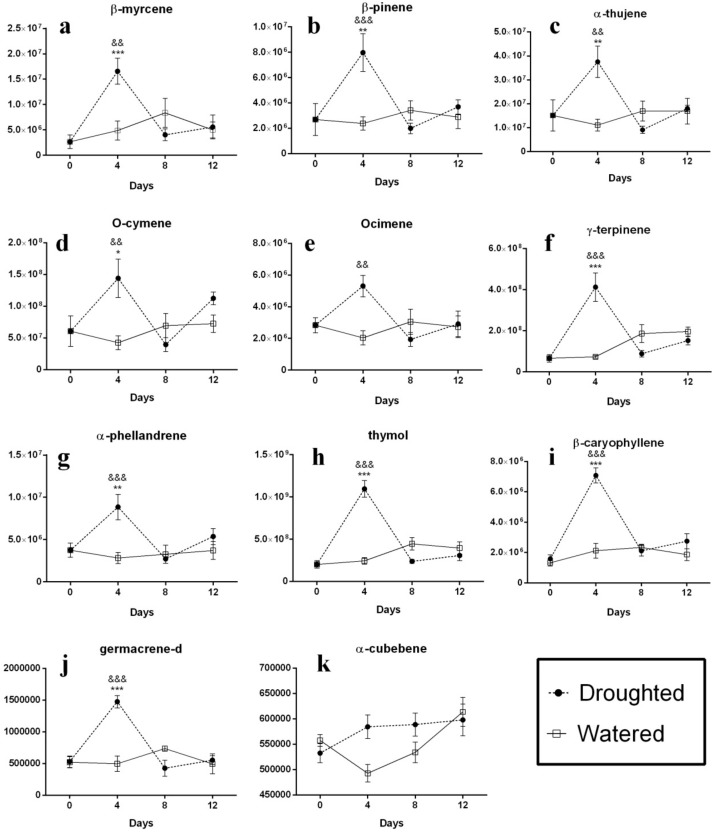
Profile of the changes of the volatile compounds in the leaves of the sensitive thyme plants during drought stress. Following water withholding, the leaves were harvested and analysis of non-polar extracts was carried out by direct infusion Fourier transform ion cyclotron resonance (DIFT-ICR) mass spectrometry. Vertical axis is the relative value of the compounds and horizontal axis the day of stress exposure. (**a**) β-myrcene; (**b**) β-pinene; (**c**) α-thujene; (**d**) O-cymene; (**e**) ocimene; (**f**) γ-terpinene; (**g**) α-phellandrene; (**h**) thymolo; (**i**) β-caryophyllene; (**j**) germacrene-d; (**k**) α-cubebene. ^*^ *p* < 0.05 vs. day 0 of Droughted group; ^**^ *p* < 0.01 vs. day 0 of Droughted group, ^***^ *p <* 0.001 vs. day 0 of Droughted group; ^&^ *p <* 0.05 Droughted vs. Watered group; ^&&^ *p <* 0.01 Droughted vs. Watered group; ^&&&^ *p <* 0.001 Droughted vs. Watered group. Data are expressed as the mean ± S.E.M. Two-way ANOVA followed by the Sidak’s multiple comparisons test was executed.

No significant decline in the net photosynthesis rate of the tolerant drought-stressed plants was observed until the 12th day and it was almost similar in the tolerant plants and the control ones (Figure 2d). After 12 days, the rate decreased in the stressed plants and it continued until the end of water deprivation (day 14) and dropped, on average, from 21 µmol^−2^ s^−1^ measured in the control plants to 13 µmol. m^−2^ s^−1^ in the stressed ones. Regarding the sensitive plants’ photosynthetic rate, it dramatically decreased from the 4th day onward.

The results totally showed that the tolerant and sensitive species behave differently under stress conditions so that their adaptations are reflected by changes in their biological compound levels and morpho–physiological characteristics. Wagner, as a drought sensitive plant, saves water while Varico3, as a drought tolerant one, shows water spender behavior.

In some plants subjected to water deficiency, stomatal conductance and net photosynthesis rates declined to about 50% compared to the control plants following drought stress treatment. It has been suggested that as drought progresses, stomata close progressively, and simultaneously, net photosynthesis decreases to main water balance and minimize stress damages and negative consequences [35,36]. The observed fast recovery of the photosynthetic apparatus in plants, such as pomegranate, after irrigation also involves some no assimilatory processes such as enhanced Mehler reaction and/or photorespiration to decompose some of the excess excitation energy and to avoid photoinhibitory damage in drought-stressed plants [37].

### 2.2. Non-Targeted Volatile Profiling of Thyme Extracts Using GC/MS

Our main goal was to investigate and compare the response of the volatiles in the drought-stressed tolerant and sensitive thyme plants (Table S1). For this end, we used a non-targeted GC/MS-based approach for simultaneously measuring a wide range of these compounds. Different compounds (range of 200–400 s of scanning) were detected with this approach, but we only discuss the major ones in the present study.

It is worth noting, however, that plants constitutively release most volatile organic compounds, such as terpenes, and their emissions occur throughout the plants’ life cycle; environmental stresses including biotic and abiotic stresses such as drought, may induce their de novo synthesis [38].

It is interesting to note that, according to the results of the calculation of Pearson correlation coefficient (r), our studied volatiles could be classified into two groups (Table S2). The first group includes the compounds β-pinene, α-thujene, ocimene, gemacrene-D and α-cubebene and the second one contains α-phellandrene, β-myrcene, O-cymene, γ-terpinene, thymol and β-caryophyllene. In each group, there is a strong positive linear correlation between every pair of metabolites while a negative linear correlation was observed between two groups. There is a distribution of both monoterpenes and sesquiterpenes into two groups.

The investigation of the different volatile contents from the drought-stressed sensitive thyme plants shows a similar pattern for their alternations compared to the control group during the stress period (Figure 2). As shown in the figure, the content of almost all volatile compounds (including β-pinene, α-phellandrene, O-cymene, γ-terpinene, α-thujene, ocimene, β-myrcene, β-caryophyelene, germacrene D and thymol) in the leaves of the stressed plants immediately increased after water deprivation with a steep slope until the 4th day and then sharply dropped between the 4th and 8th days. Although a slight increase was observed for compounds from the 8th day to the end of the stress period, their levels were lower than those from the control group. The compound α-cubebene was the only exception in this group and revealed a different pattern of the alternations following the stress imposition (Figure 2k). Although the level of this volatile in the leaves of the drought-stressed plants was always more than that in the control group, the monitoring of its alternations revealed that following an increase in its intensity from days 0 to 4, its content was retained unchanged until the last day of the stress imposition period.

**Figure 3:**
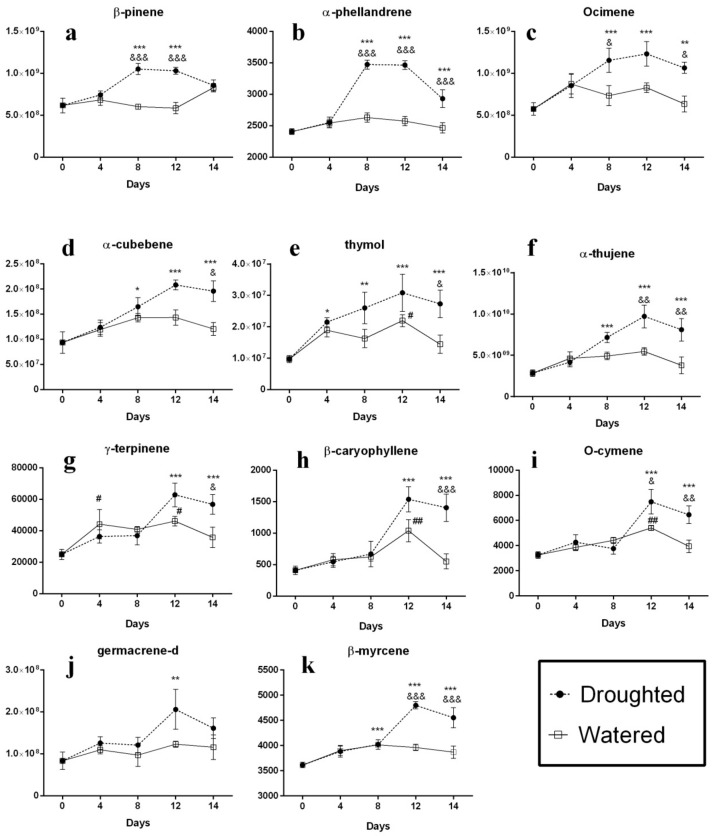
Profile of the changes of the volatile compounds in the leaves of the tolerant thyme plants during drought stress. Following water withholding, the leaves were harvested and analysis of non-polar extracts was carried out by direct infusion Fourier transform ion cyclotron resonance (DIFT-ICR) mass spectrometry. Vertical axis is the relative value of the compounds and horizontal axis the day of stress exposure. (**a**) β-pinene; (**b**) α-phellandrene; (**c**) ocimene; (**d**) α-cubebene; (**e**) thymolo; (**f**) α-thujene; (**g**) γ-terpinene; (**h**) β-caryophyllene; (**i**) O-cymene; (**j**) germacrene-d; (**k**) β-myrcene. ^*^ *p* < 0.05 vs. day 0 of Droughted group; ^**^ *p* < 0.01 vs. day 0 of Droughted group, ^***^ *p* < 0.001 vs. day 0 of Droughted group, ^##^ p < 0.01 vs. day 0 of Watered group; ^###^ *p* < 0.001 vs. day 0 of Watered group; ^&^ *p* < 0.05 Droughted vs. Watered group; ^&&^ *p* < 0.01 Droughted vs. Watered group; ^&&&^ *p* < 0.001 Droughted vs. Watered group. Data are expressed as the mean ± S.E.M. Two-way ANOVA followed by the Sidak’s multiple comparisons test was executed.

Taken together, while the analysis of the volatile compounds emitted by the sensitive thyme plants displayed a similar pattern for almost all of them, the comparison of the pattern of changes in the volatile compounds’ composition in the tolerant control and drought-stressed plants revealed that these changes can be classified into three separate groups as follows (Figure 3). In the first group, a significant increase was observed in the content of the compounds β-pinene, α-phellandrene and ocimene after the 4th day drought imposition in the stressed thyme plants compared to the control group, and this increase continued until the 8th day after stress for all three compounds (Figure 3a–c). The highest increase was observed for α-phellandrene and after 8 days, its concentration was retained in a steady-state level until the 12th day. However, the volatiles β-pinene and ocimene showed a slight decrease and increase, respectively, in their intensities between days 8 and 12. A relatively severe decrease was finally observed in the emission of all three volatiles in the last period of drought stress (from day 12 to 14). In the second class (Figure 3d–f), we can see an increasing rate of the concentration of the volatile compounds including the compounds α-cubebene, thymol and α-thujene, immediately after the stress imposition that lasts until the 12th day. After, a declining pattern in the intensities of the mentioned volatile levels until the last day of the stress period is observed. While the increase in the amounts of the compounds α-cubebene and thymol is relatively mild compared to the control group (Figure 3d,e), the emission of α-thujene shows a significant increase in comparison with the two other compounds as well as the control group (Figure 3f). The third category’s volatiles (γ-terpinene, β-caryophyelene, O-cymene, β-myrcene and germacrene D) show a slightly different pattern in their compositions during the stress period compared to the two other groups (Figure 3g–k). As seen from the figure, most changes occur in their contents into the two last intervals (days 8–12 and 12–14, respectively) as a sever increase on the 8th day to 12th day and then a decrease between days 12–14. The pattern of these volatiles’ changes on days 0–8 is variable. While the emission of the compounds β-caryophyelene and β-myrcene is similar to the control groups (Figure 3h,k) and their curves are well superimposable, the volatiles germacrene D and γ-terpinene show increased and decreased levels (Figure 3j,g), respectively, in this period. In the case of O-cymene, a slightly increased amount followed by a decreased amount was observed on days 0–4 and 4–8 after drought imposition, respectively, compared to the control plants (Figure 3i).

Totally, according to our results, the tolerant thyme plants show a prominent adaptation to drought stress. The levels of the mentioned volatiles have increased in the green leaves of the tolerant thyme plants in response to stress, suggesting a possible role of these compounds in drought stress response in these plants. Indeed, these compounds may act as signaling molecules in response to damages induced by water deprivation. However, the interesting question of what their physiological importance in thyme plants under water stress is should be analyzed by further studies. According to our results, however, there are some fluctuations in the patterns of their content alterations. Indeed, they display increased levels compared to the control group and this trend lasts until the end of the stress period (14th day). Their various increasing or decreasing contents at the different stress intervals likely indicate their different contributions to the stress adaptation during the stress period. Another noticeable fact is that it looks like the tolerant plants’ adaptations to the stress improve over the time what is not observed in the case of the sensitive plants. It means that the intensity of all desired compounds in the sensitive plants increase transiently at early stages of the stress treatment (until 4th day). After, during the last stress interval (days 8 to 12) a sharp decrease of volatile molecules is observed. Indeed, most of them have decreased levels compared to the control group that means they could not overcome to the stress conditions and lose their survival. In one report, Catola et al. evaluated the effects of drought stress on the volatile organic compounds extracted from the leaves of pomegranate plants. Their results showed that among 12 volatile compounds detected in the leaf profiles, the compound trans-2-hexenal displayed a significant increase in water stressed and recovered leaves indicating a possible role of the oxylipin pathway in the response of pomegranate plants to water stress [39].

### 2.3. Integrative Analysis of Volatile Compounds in the Tolerant and Sensitive Thyme Plants

**Figure 4:**
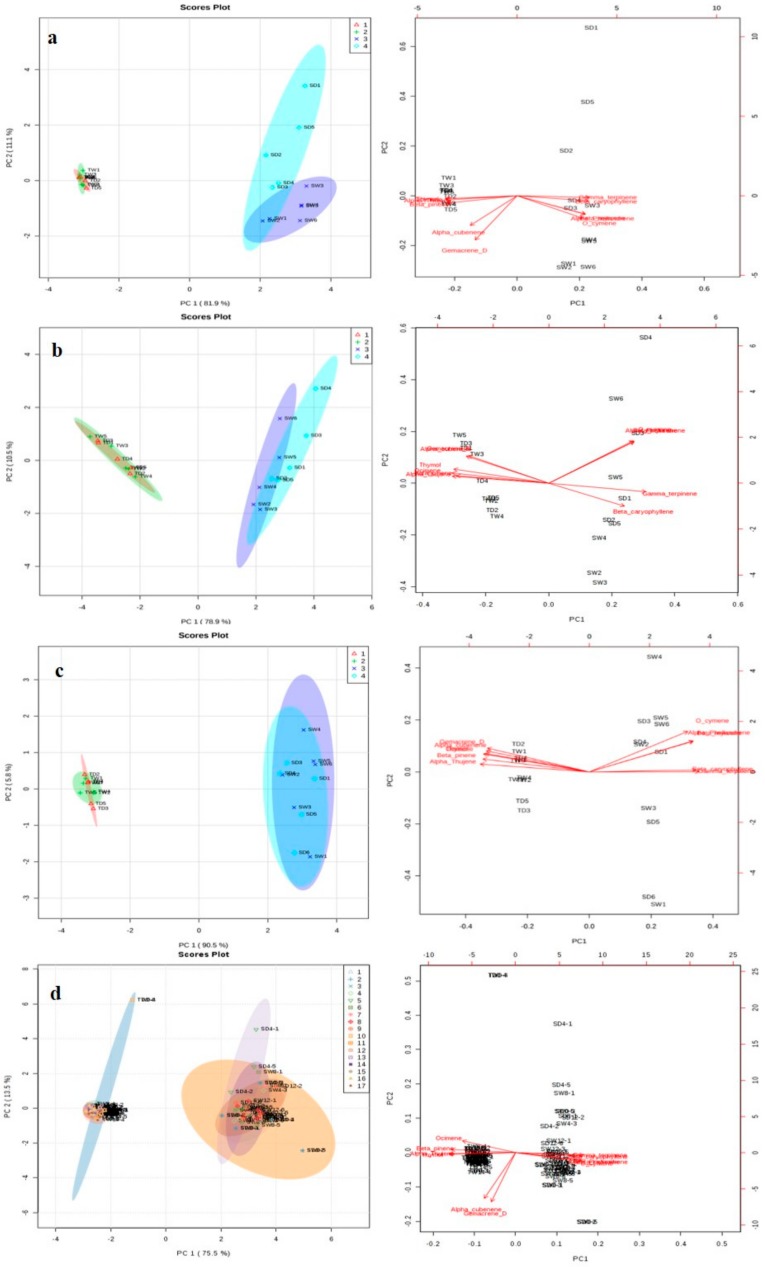
Principal component analysis (PCA) and partial least squares-discriminate analysis (PLS-DA) score plots for the volatile compounds in the tolerant and sensitive thyme leaves under control and drought stress conditions. Score plots for the tolerant watered (TW), tolerant drought-stressed (TD), sensitive watered (SW) and sensitive drought-stressed (SD) leaves after four (**a**), 8 (**b**) and 12 (**c**) days of water limitation. (**d**) The total plots (days 0–14). The numbers present the corresponding sample numbers.

Principal Component Analysis (PCA) was used to differentiate between the metabolite profiles of the treated and untreated sensitive and tolerant thyme plants and to highlight the main compounds responsible for this differentiation. Several statistical values such as variable importance on projection (VIP), the regression coefficient, and loading weight that are utilized for metabolite identification can be provided by this technique. This technique allows us to construct visually interpretable and low-dimensional score plots from a large dataset. In fact, this statistical method is applied to indicate differences and similarities between individuals and pattern recognition [40,41]. Reasonable classification between the categories was observed in all experimental days and changes induced by drought stress in the levels of various volatile compounds were associated with water deprivation. (Figure 4). Figure 4a shows the volatile compounds contents in the experimental plants 4 days after stress exposure, sensitive and tolerant plants were well grouped by first principal component (PC1) which could explain 81.9% of the total variation, whereas PC2 allocating 11.1% of the total variation. On day 8 (Figure 4b), the detected compounds reveals the first component with 78.9% of the total variation classified plants to sensitive and tolerant, while the PC2 showed 10.5% of variation. Figure 4c illustrates the results after 12 days of the experiment with the PC1 and PC2 explaining 90.5 and 5.8% of the total variation, respectively. According to the results, it seems that the compounds O-cymene, α-phellandrene, β-caryophyelene and γ-terpinene play crucial roles in-group discrimination between sensitive and tolerant populations.

**Figure 5:**
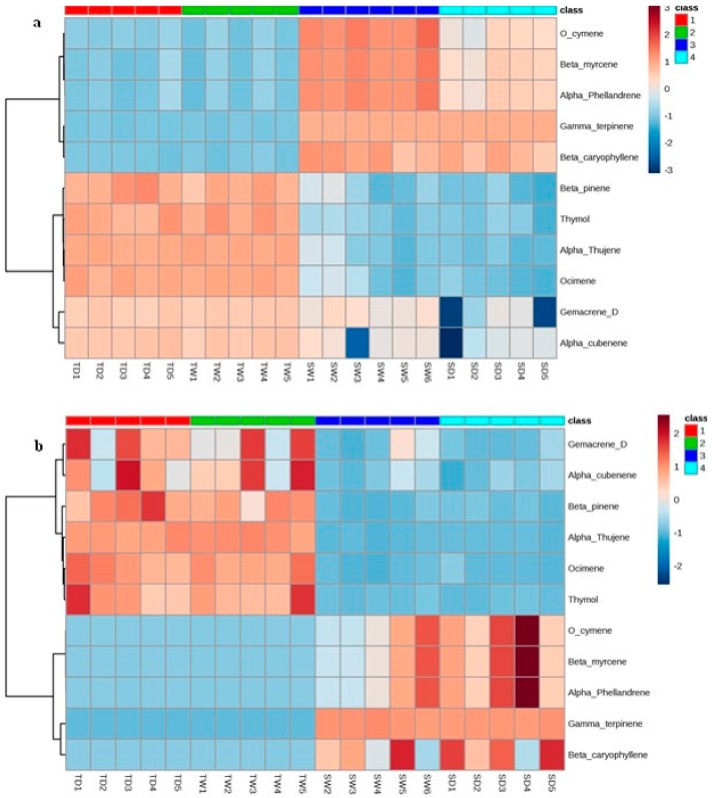

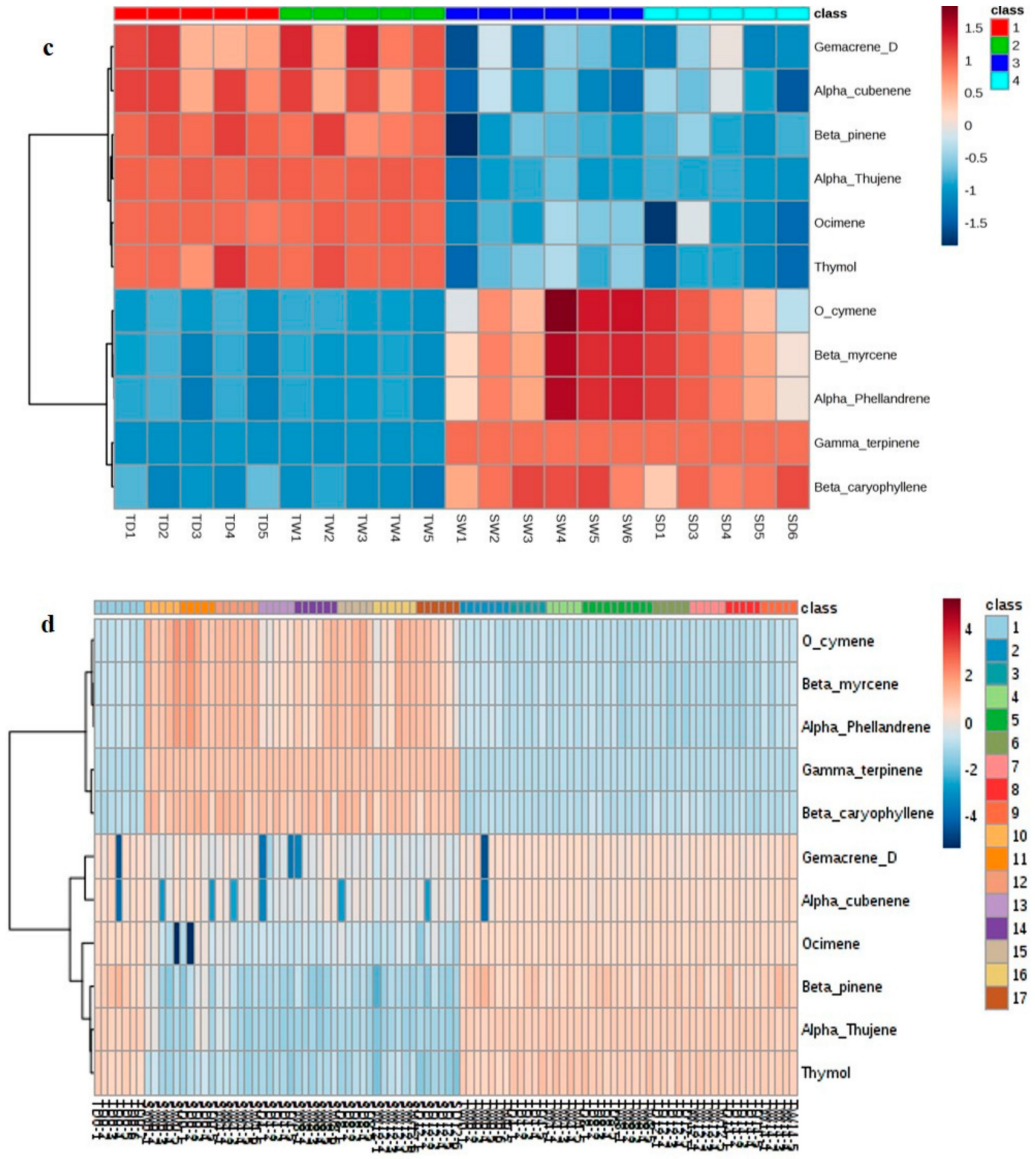
The hierarchical clustering and heat map of the data. The peaks were divided into given groups (TD, tolerant drought-stressed; TW, tolerant watered; SD, sensitive drought-stressed; SW, sensitive watered) after four (**a**), 8 (**b**) and 12 (**c**) days of water limitation. (**d**) The total heat map (days 0–14). The numbers represent the corresponding sample numbers and different colors are indicative of the relative abundance of each volatile in different condition.

Data visualization was performed using heat maps [40,42,43]. Heat map analysis showed that most volatiles in the TD and SD groups have been affected by water deprivation, with a significant difference in their changing patterns (Figure 5). Based on the data analysis results, the volatile compounds O-cymene, α-phellandrene, β-caryophyelene, γ-terpinene and β-myrcene have been up-regulated in the sensitive plants while the compounds β-pinene, thymol, α-thujene, ocimene, germacrene D and α-cubebene have been down regulated in this population in all stress periods (Figure 5a–d). Interestingly the opposite trend is observed in the tolerant species.

**Figure 6:**
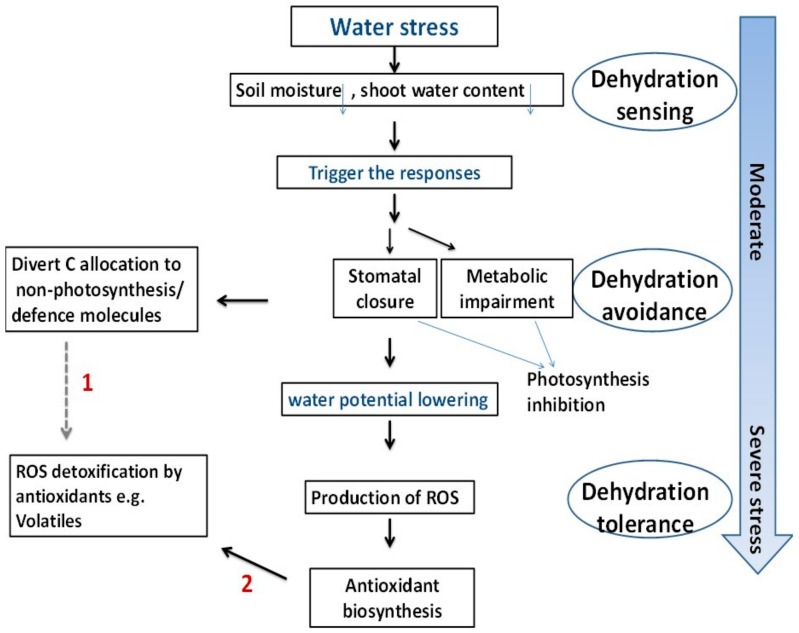
The suggested adaptation mechanisms to drought stress in thyme plants. (1) Pathway illustrates carbon diversion from photosynthesis to VOCs and (2) depicts ROS scavenging by non-enzymatic antioxidants.

Finally, Figure 6 depicts our suggested adaptation mechanisms to drought stress in thyme plants. As shown in the figure, the stressed plant diverts its carbon allocation from photosynthesis to produce defense molecules such as volatile compounds. Whilst under normal conditions, a plant allocates about two percent of the carbon pool for synthesis of these compounds.

## 3. Materials and Methods

### 3.1. Plant Material and Morpho-Physiological Parameters

Wagner as a susceptible population of thyme plants (*Thymus vulgaris*) Varico3 as drought tolerant plants have been used in this experiment, which seed, were provided by Semilas Silvestris Company in Spain. Selection process of these population were based upon physiological experiments published previously [44,45].

**Figure 7:**
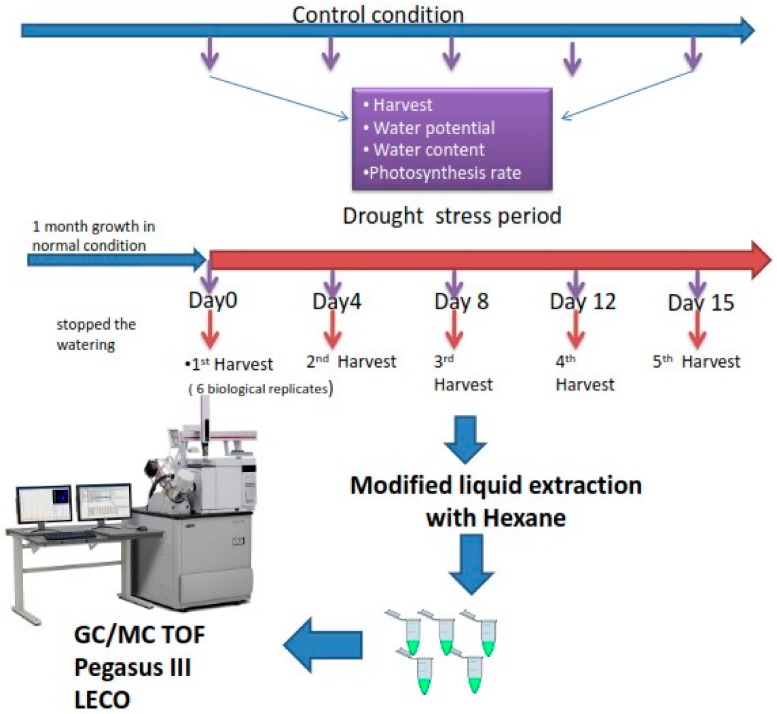
Experimental design to explore the volatile composition alterations over drought stress period using combined physiological and non-targeted volatilome profiling.

Seeds were planted in soil mixture of 4:1 ratio of peat: perlite. Growth room condition was sixteen hours light and eight hours darkness in temperature of twenty-two degrees Celsius. Two groups of pots including watered plants (weekly watering) and droughted plants kept growing until one-month-old plants. Watering stopped for droughted plants group to impose stress. Physiological and morphological traits were measured in four-day intervals during the stress period (Figure 7).

For physiological traits, soil moisture, water content, water potential and shoot dry weight were measured at each time point of stress period. HH2 Moisture meter model SM300 by Delta-T Devices Ltd. were used to identify soil moisture levels. Soil moisture content was identified using with 2.5% accuracy. Pressure cylinder Skye Company Model SKPM 1400 was used to identify shoot water potential. To record stomatal conductance (mol CO_2_.m^−2^ s^−1^) and net photosynthesis rate (μmol CO_2_.m^−2^ s^−1^), portable gas exchange measuring system (LCI BioScientific, Great Amwell, UK) was applied.

### 3.2. Sampling and Extraction Procedure

Harvesting of the similar ages leaf samples from the sensitive and tolerant thyme plants was began from water withholding until the 15th day stress and sampling interval was 4 days. Liquid nitrogen was used to flash-frozen of the samples and they were then weighed and stored at −70 °C until used. Biological replicates were six per time point in each sampling and their fresh weights ranged from 30–100 mg. Extraction was carried out using a modified extraction method. For this end, the frozen samples were immediately transferred into liquid nitrogen and then ground by pestle in microfuge tubes, and finally placed back in liquid nitrogen tubes. Following weighing the samples, 1 mL hexane containing 10 ng µL^−1^ benzyl acetate (as the internal standard) was added to each microtube. The tubes were then vortexed for fifteen seconds and centrifuged at 13,000 rpm for ten minutes. The resultant supernatants were then collected and poured into 1.5 mL brown glass vials.

### 3.3. GC/MS Analysis of Volatile Compounds

One µL of the extracts was injected into the GC/MS-TOF (gas chromatography time of flight) (Pegasus III, Leco, St. Joseph, MI) using the auto sampler. Compounds’ separation was carried out on a capillary column (DB-5MS UI, 10 m long, 0.180 mm id and 0.18 µm film thickness (Hewlett Packard, Palo Alto, CA)) for three minutes at 40 °C and then raised at 30 °C min^−1^ to 250 °C and hold for two minutes. The used carrier gas was helium and its flow rate was set to 3 mL min^−1^ (for two minutes) and 1.5 mL min^−1^ afterwards. The mass spectrometry setting was prepared for generating a mass spectrum at 70 eV with a 90 s solvent delay at 1597 eV at 20 scans/second. The selected mass range was 50 to 350 atomic mass units (AMU).

### 3.4. Volatile Identification and Data Processing

Volatiles’ identification was performed by either automatic recognition using the spectral library of the instrument software (LECO Chroma TOF version 1.00 Pegasus driver 1.61) or comparative study of the previously published data. The instrument software was used to peaks’ identification and the volatile compound reference [46] and www.pherobase.com were applied for data confirmation. In the case of unknown peaks, Kovat Indexes (KI) were calculated based on Retention Time. Below formula was used for calculating the KI value for each compound:

KI (x) = 100 × ([log RT (x) − log RT (alkane on the left)] − log RT (alkane on the left)] × [number of carbon atoms of alkane on left]).(1)

To confirm the peaks’ identification, the calculated values were compared with the reference (Adams, 2007). For data quantification, peak areas were corrected by the internal standard (benzyl acetate), sample weight and 11 external standards including α-terpinene, β-myrcene, α-phellandrene, α-humulene, β-phellandrene, γ-terpinene, cis, β-ocimene, linalool, carvacrol, terpinolene and thymol as described previously [47]. Six technical replicates were run for each sample by GC/MS.

### 3.5. Chemometrics Analyses and Data Processing

The probabilistic quotient normalization (PQN) method was used for data set normalization and reduction of the effects of extreme peak intensities [42]. Then, the K-nearest neighbor (KNN) imputation technique was applied to fill in the missing data [43,48]. The grouped data were finally transformed using the generalized log transformation (GLog) method [49] in order to removing the domination of the highest intensity peaks by stabilizing the whole variance. Principal component analysis (PCA) was then performed using JMP software. The results display any structure correlated to the data. Actually, data are converted into a simple visual format by PCA. Heat map as a two-way cluster analysis was carried out by JMP to classify compounds and individuals simultaneously. In fact, this technique rearranges rows and columns to create a statistical trend from the dataset.

### 3.6. Statistical Analysis

Two-way ANOVA with Šidák multiple comparison tests was performed for analysis of the effects of drought stress on the physiological parameters and of the changes of the volatiles compounds. Statistical analyses were performed by GraphPad Prism 6 software, (San Diego, CA, USA). Data are presented as the means ± S.E.M, with the statistical significance level set at *p* < 0.05.

## 4. Conclusions

The impact of water deficient stress on the levels of volatile compounds and physiological parameters over 30 days in sensitive and tolerant thyme plants (*T. vulgaris* Var. Wagner and *T. vulgaris* Var. Varico3, respectively) was investigated. The sampling and data recording were carried out at four-day interval. The obtained results revealed that while the tolerant and sensitive plants qualitatively and quantitatively react differently to physiological parameters, the most stress-induced changes on the plants’ physiological traits occurred in the photosynthetic rates, where the tolerant plants maintained their photosynthesis similar to the control ones till the 8th day of the drought stress period. In fact, it seems that stress could affect the plants’ photosynthetic activity from the 12th day onward. The sensitive thyme plants were severely affected by the stress since early days of stress (4th day). It seems that considering the lack of adaptation strategies in these plants, declined photosynthetic activity (50% less than the control ones) remained till the end of water depletion. At the metabolic level, the measurement of volatile compound content in the sensitive plants showed the same pattern for almost all of them; the analysis of the volatile compounds’ levels in the tolerant drought-stressed plants showed that these changes could be grouped into three different classes.

The compounds β-pinene, α-phellandrene and ocimene that were located in the first group showed an increase–constant–decrease trend while the second category including α-cubebene, thymol and α-thujene followed an increase–slight decrease pattern. Finally, the trend of the third group which was composed of the volatile compounds γ-terpinene, β-caryophyelene, O-cymene, β-myrcene and germacrene D was a sharp increase in their levels from the 8th to 12th days; this was then followed with a decrease until the end of the stress period. PCA analysis revealed that the most determinant compounds involved in drought stress adaptation were α-phellandrene, O-cymene, γ-terpinene and β-caryophyelene. Totally, it seems that while the sensitive plants trade-off between growth and defense, the tolerant ones simultaneously activate their stress response mechanism and continue their growth.

## Supplementary Materials

The following are available online.
