# Supplementary material for: Variation in Terpene Profiles of Thymus vulgaris in Water Deficit Stress Response
Source: Molecules. 2020 Feb 28;25(5):1091. doi: 10.3390/molecules25051091 (PMC7179171; doi:10.3390/molecules25051091)
Supplement: Supplementary file 1 [file molecules-25-01091-s001.pdf]

Table S1. Major volatile components detected in thyme populations by modified liquid extraction method from fresh leaves.

No	Compound	Formula	Major Ion	RT**	KI***
1	Beta-Pinene	C10H16	93	193	980
2	Beta-Myrcene	C10H16	41,93, 69	217	991
3	Alpha- Phellandrene	C10H16	93	225	1005
4	Alpha-Thujene	C10H16	93	233	931
5	*p*-Cymene	C10H14	119	240	1026
6	Alpha-Cubebene	C15H24	161	410	1351
7	Ocimene	C10H16	93	242	1050
8	Gamma-Terpinene	C10H16	93	260	1062
9	Benzyl acetate*	C9H10O2	108	320	1163
10	Thymol	C10H14O	135	355	1290
11	Beta-Caryophyllene	C15H24	93,133	383	1418
12	Germacrene D	C15H24	161,105	407	1480

* Benzyl acetate was used as internal Standard; ** Retention times are based on the GC-MS under the conditions mentioned in materials and methods; *** Reference for KI: Adams,R.P. (2007) on DB-5 column.

Supplementary Table S2. Summary of Pearson correlation coefficient values between pairwise metabolites.

	Pearson correlation coefficient										
	β-pinene	α-thujene	Ocimene	Gemacrene-D	α-cubebene	α-phellandrene	β-myrcene	O-cymene	γ-terpinene	Thymol	β-caryophyllene
β-pinene	1	00.870**	0.906**	0.870**	0.925**	−0.684**	−0.552**	−0.669**	−0.614**	−0.611**	−0.657**
α-thujene	0.870**	1	0.929**	0.871**	0.909**	−0.587**	−0.483**	−0.573**	−0.528**	−0.514**	−0.563**
Ocimene	0.906**	0.929**	1	0.850**	0.921**	−0.663**	−0.539**	−0.646**	−0.592**	−0.583**	−0.637**
Gemacrene-D	0.870**	0.871**	0.850**	1	0.852**	−0.604**	−0.496**	−0.588**	−0.540**	−0.535**	−0.591**
α-cubebene	0.925**	0.909**	0.921**	0.852**	1	−0.673**	−0.562**	−0.641**	−0.602**	−0.601**	−0.663**
α-phellandrene	−0.684**	−0.587**	−0.663**	−0.604**	−0.673**	1	0.845**	0.854**	0.833**	0.864**	0.886**
β-myrcene	−0.552**	−0.483**	−0.539**	−0.496**	−0.562**	0.845**	1	0.808**	0.854**	0.892**	0.868**
O-cymene	−0.669**	−0.573**	−0.646**	−0.588**	−0.641**	0.854**	0.808**	1	0.845**	0.835**	0.805**
γ-terpinene	−0.614**	−0.528**	−0.592**	−0.540**	−0.602**	0.833**	0.854**	0.845**	1	0.966**	0.870**
Thymol	−0.611**	−0.514**	−0.583**	−0.535**	−0.601**	0.864**	0.892**	0.835**	0.966**	1	0.932**
β-caryophyllene	−0.657**	−0.563**	−0.637**	−0.591**	−0.663**	0.886**	0.868**	0.805**	0.870**	0.932**	1

** Correlation is significant at the 0.01 level (2-tailed).
